# The disequilibrium in the distribution of the primary health workforce among eight economic regions and between rural and urban areas in China

**DOI:** 10.1186/s12939-020-1139-3

**Published:** 2020-02-26

**Authors:** Yueyue Wang, Yuyang Li, Shangren Qin, Yuanfeng Kong, Xiyang Yu, Keqiang Guo, Jiayu Meng

**Affiliations:** 1 School of Medicine, Hangzhou Normal University, Hangzhou, 310036 Zhejiang China; 2 The First Affiliated Hospital of Wenzhou Medical University, Wenzhou, 325000 Zhejiang China

**Keywords:** Equity, Primary healthcare, Health workforce, Distribution, New medical reforms

## Abstract

**Background:**

Equity is one of the major goals of China’s new medical reforms launched in 2009. This study aimed to analyze the disequilibrium in primary health care (PHC) workforce among various economic zones in China and to compare the fairness between urban and rural areas since the implementation of the new medical reforms.

**Method:**

According to China’s 11th Five-Year Plan, China is divided into eight economic regions. The data of this study were obtained from China Statistical Yearbook 2009–2016. The Atkinson index was used to depict the trend of PHC workforce fairness; the Gini coefficient was used to compare the fairness of workforce distribution between urban and rural areas; the health resource agglomeration degree was used to analyze the distributional equity of the workforce in the eight regions; and the Theil Index was used to compare the fairness of urban and rural workforce distribution across eight regions.

**Result:**

The Atkinson index indicated that the equity of the entire PHC workforce allocation had generally improved during the new medical reforms; the Gini coefficient indicated that the fairness of the entire workforce allocation had improved in cities, but only the nurse allocation became fairer in rural areas. The agglomeration degree and the Theil index indicated that the fairness gaps across the eight regions were still large. These analyses differed from previous studies where China was divided into western, central and eastern regions. In what was previously defined as eastern region, the northeast was under-resourced, while the eastern coastal areas were observing a resource surplus. In western region, we found that the fairness in the northwest was significantly worse than southwest.

**Conclusion:**

In China, the distribution of healthcare workforce has been improved with continuous effort. The gaps in the distribution of PHC workforce across different economic regions and between urban and rural areas are still large, with different regions facing different problems. The government should consider the population and geographical factors in allocation of PHC workforce, especially nurses.

## Background

In 2014, China’s total health expenditure was approximately 520 billion dollars, accounting for 5.5% of the annual GDP [1]. Although this proportion was lower in comparison to most high-income countries, it was higher than many middle-income countries.

Meanwhile, China actively launched multiple waves of medical reforms since 2009 [2], during which a fundamental medical and health system covering urban and rural areas was established with the aim of delivering health services that are “safe, convenient, inexpensive and effective”.

When evaluating the advancement of medical reforms, the quality of primary healthcare (PHC) system has been a major concern. Among emerging problems that restrict the development of PHC, insufficiency and imbalance pose two important aspects. Especially, many studies have shown that an equitable PHC system not only affects residents’ health level, but also plays an important role in eliminating socioeconomic disparities in healthcare service utilization [3, 4]. With the government’s intensive effort in developing fundamental healthcare undertakings, the residents’ acceptance of PHC services and their utilization of primary health settings have been increasing by year [5]. However, the unfairness of PHC services still deserve public attention [6].

Primary health workforce refers to the providers of primary health services among all health resources, which directly influences the fair use of health services. Several previous studies investigated the distribution of primary health resources in China. Tao [7] compared the resource distribution between primary healthcare institutions and hospitals; Yue [8] studied the fairness and efficiency of health resource allocation in China. Their work primarily focused on the distribution of entire PHC workforce across western/central/eastern regions. In this study, we further inspected the allocation of physicians, nurses and health professionals as three separate indicators of PHC workforce, and analyzed distributional fairness against eight economic zones, according to China’s 11th Five-Year Plan. We believe that this division better aligns with current policy environment and socio-economic situation [9–12], In consideration of rapid course of urbanization, we analyzed the inequity of PHC workforce distribution between urban and rural areas.

The study aimed to use multiple valid methods to analyze the inequity of PHC workforce distribution against the new division of economic zones, and to analyze the disparity between urban and rural areas. The results of the study might be helpful to determine the effects of the new medical reforms and to provide a reference for the government during its decision-making processes targeting to establish high-quality primary health services that cover the entire population.

## Methods

### Data sources

The geographic data were taken from China Statistical Yearbook 2017. Due to inconsistencies of data standards, this study included 31 provinces and municipalities in China, while excluding Hong Kong, Macao and Taiwan. Urban areas include municipalities and prefecture-level cities, and rural areas include county and county-level cities. The number of health professionals, physicians and nurses were obtained from the China Health Statistics Yearbook 2010–2017. A series of longitudinal data was used to examine the change of distributional equity in the country from 2009 to 2016.

### Setting

**Fig. 1:**
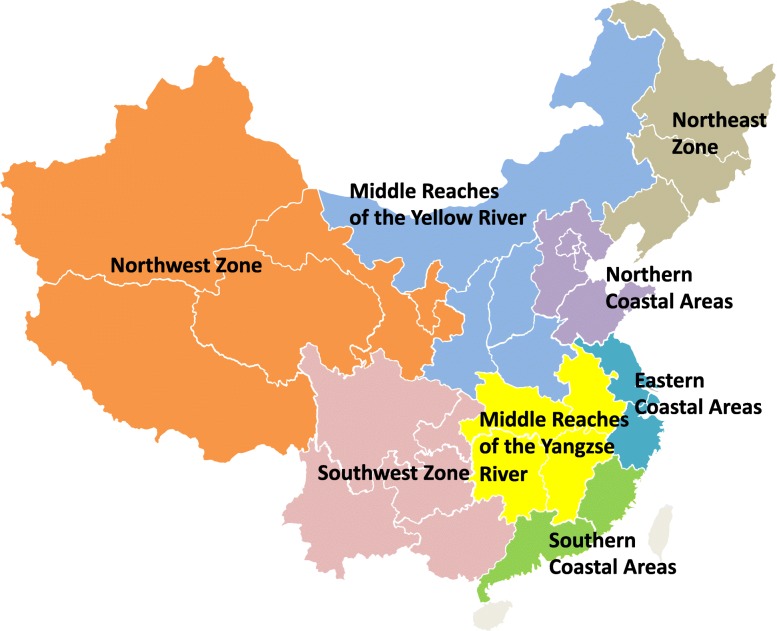
The distribution of eight economic zones in China

The 11th Five-Year Plan, based on population, resources, ecology and economic factors, China was divided into eight economic zones: the northeast zone, including Liaoning, Jilin and Heilongjiang; the northern coastal areas, including Beijing, Tianjin, Hebei and Shandong; the eastern coastal areas, including Shanghai, Jiangsu and Zhejiang; the southern coastal areas, including Fujian, Guangdong and Hainan; the middle reaches of the Yellow River, including Shanxi, Henan, Shanxi and Inner Mongolia; the middle reaches of the Yangtze River, including Hubei, Hunan, Jiangxi and Anhui; the southwest zone, including Yunnan, Guizhou, Sichuan, Chongqing and Guangxi; and the northwest zone, including Gansu, Qinghai, Ningxia, Tibet, and Xinjiang. According to the division of the eight economic zones, we created the map by using the mapping website, the geographical features are shown in Fig. 1. Social and economic features of the eight economic regions (Table 1) are shown as below.

**Table 1:** Basic information on China’s eight economic zones

Regions	Population(*10000)	Area (km2)	GDPPC (YUAN)	The per capita total health expenses (YUAN)
Northeast	10,910	78.81	47,947.02	3003.73
Northern coastal	21,152	36.98	66,953.86	3474.51
Eastern coastal	16,009	21.09	93,720.66	4244.34
Southern coastal	15,790	33.53	70,978.97	3009.74
Middle reaches of the Yellow River	19,547	171.19	46,616.10	2707.29
Middle reaches of the Yangtze river	23,495	70.44	45,126.58	2449.19
Southwest	24,474	135.91	38,853.17	2479.69
Northwest	6607	413.11	35,783.11	3169.24
Total	137,984	961.06	55,942.77	2980.8

### Indicators

Three groups of indicators were chosen to measure inequity: health professionals, physicians and registered nurses. According to the standards used in Health Statistics Yearbook, health professionals included physicians, nurses, pharmacists, technicians, and health inspectors.

Primary health institutions included community health service organizations (CHOs) in urban areas and the township health centers (THCs) and village clinics in towns and villages. We used the results of distributional fairness of the health workforce between the CHOs and THCs to represent the fairness of the PHC workforce distribution in urban and rural areas.

### Measurements of inequity

In this study, we used four methods to measure inequity. First, the Atkinson index was used to examine the change in the national distributional equity. Second, the Gini coefficient was used to compare the fairness of the PHC workforce distribution nationally between the urban and rural areas. Third, the health resource agglomeration degree was used to analyze the distributional equity of the workforce in the eight regions. Fourth, the Theil Index was used to compare the fairness of the urban and rural workforce distribution in the eight regions.

### Atkinson index

The Atkinson index was originally an index that measures the notion of social welfare norms in income distribution unfairness [13]. It is mainly used to measure the equity of income and has gradually become adopted in the field of health resources. First, an equivalent number of sensitive average health resources is calculated (ye), which is the level of per capita health resources, that if equally distributed, would give the same level of social welfare as the actual distribution of health resources. The index is given by the following formula:

$$ {y}_e=\left[\frac{1}{n}\sum \limits_{i=1}^n{s}_i{\left({y}_i\right)}^{1-\varepsilon}\right]\frac{1}{1-\varepsilon } $$

$$ \mathrm{A}=1-\frac{y_e}{\mu } $$

(A) is the Atkinson index; i is region i; (si) is the proportion of the population/geographic area of region I in the national population/geographic area; (yi) is per thousand hectares; and ε is used to assume the level of inequality aversion, which reflects the strength of society’s aversion to inequality. When the value of ε rises, relatively more weight is attached to transfers at the lower end of the distribution and relatively less weight is attached at the upper end. The coefficient ε in the present study was set at 0.5 to calculate the Atkinson index [14–16]. The Atkinson index ranges from 0 to1, the more equal the distribution, the value of it is more close to 1.

### Gini index

The Gini coefficient has been identified as a superior tool for measuring inequity [17]. The Gini coefficient ranges from 0 to 1, and the closer this value is to 0, the better the fairness. A G < 0.2 indicates that resource allocation is absolutely fair; 0. 2 ≤ G < 0.3 indicates that resource allocation is relatively fair; 0.3 ≤ G < 0.4 indicates that resource allocation is absolute equal; 0.4 ≤ G < 0.5 indicates that resource allocation is relatively unfair; and G ≥ 0.5 indicates that resource allocation is highly unfair and that the gap is wide [18, 19]. The Gini coefficient is calculated as follows:

$$ \mathrm{G}=\sum \limits_{i=1}^{n-1}\left({x}_i{y}_{i+1}-{x}_{i+1}{y}_i\right), $$

where *x*_*i*_ is the proportion of the population in the region to the total population, *y*_*i*_ is the proportion of PHC workforce in the region relative to the total PHC workforce, and n is the number of regions.

### Theil index

The Theil index is decomposable in the analysis of fairness. It can be divided into several parts to analyze the fairness of the whole. Similarly, regional differences and interregional differences can be calculated according to the formula. The Theil index is calculated as follows:

$$ \mathrm{T}=\sum \limits_{i=1}^n{p}_i Ln\left(\frac{p_i}{y_i}\right), $$

where *p*_*i*_ is the proportion of the population in the region relative to the total population, *y*_*i*_ is the proportion of the PHC workforce in the region relative to the total PHC workforce, n refers to the number of provinces in the economic zone, and i refers to the ith province in the economic zone.

T can be divided into Tintra and Tinter, and their calculations are as follows:

$$ {\displaystyle \begin{array}{l}\mathrm{T}={\mathrm{T}}_{intra}+{\mathrm{T}}_{inter}\\ {}{\mathrm{T}}_{intra}=\sum \limits_{g=1}^k{P}_g{T}_g\\ {}{\mathrm{T}}_{inter}=\sum \limits_{g=1}^k{p}_g Ln\left(\frac{p_g}{y_g}\right)\end{array}} $$

where Pg is the proportion of the population of each economic zone relative to the total population of the country, Yg is the proportion of PHC workforce of each economic zone relative to that of the whole country, k refers to the number of economic zones, and g refers to the gth economic zone of the country [20].

### Agglomeration degree

The agglomeration degree refers to the concentration degree of a certain production factor in a certain region relative to a larger region [21, 22]. Industrial agglomeration is mainly used in the field of economics to measure the concentration degree of a certain industry in different regions [23–25].

The population agglomeration degree (PAD) is a new concept in which the agglomeration degree is used in demographic research to reflect the indicator of the degree of population agglomeration of a region relative to the whole country. In this study, it shows the proportion of the national population in a region that occupies 1% of the national area [26, 27], and the calculated formula is as follows:

$$ {\mathrm{P}\mathrm{AD}}_{\mathrm{i}}=\frac{\left(\raisebox{1ex}{${\mathrm{P}}_{\mathrm{i}}$}\!\left/ \!\raisebox{-1ex}{${\mathrm{P}}_{\mathrm{n}}$}\right.\right)\times 100\%}{\left(\raisebox{1ex}{${\mathrm{A}}_{\mathrm{i}}$}\!\left/ \!\raisebox{-1ex}{${\mathrm{A}}_{\mathrm{n}}$}\right.\right)\times 100\%}=\frac{\raisebox{1ex}{${\mathrm{P}}_{\mathrm{i}}$}\!\left/ \!\raisebox{-1ex}{${\mathrm{A}}_{\mathrm{i}}$}\right.}{\raisebox{1ex}{${\mathrm{P}}_{\mathrm{n}}$}\!\left/ \!\raisebox{-1ex}{${\mathrm{A}}_{\mathrm{n}}$}\right.} $$

PADi is the population agglomeration degree of a certain region I, Pi is the population quantity of a certain region I, Ai is the land area of a certain region I, An is the land area of the country, and Pn is the total population of the country. In recent years, the concept of agglomeration degree has been gradually introduced into the field of health as a new indicator to evaluate the fairness of the allocation of health resources [28, 29]. It is defined as the proportion of health resources in a certain region that occupies 1% of the land area of the country. The calculation formula is as follows:

$$ {\mathrm{HARD}}_{\mathrm{i}}=\frac{\left(\raisebox{1ex}{${\mathrm{HR}}_{\mathrm{i}}$}\!\left/ \!\raisebox{-1ex}{${\mathrm{HR}}_{\mathrm{n}}$}\right.\right)\times 100\%}{\left(\raisebox{1ex}{${\mathrm{A}}_{\mathrm{i}}$}\!\left/ \!\raisebox{-1ex}{${\mathrm{A}}_{\mathrm{n}}$}\right.\right)\times 100\%}=\frac{\raisebox{1ex}{${\mathrm{HR}}_{\mathrm{i}}$}\!\left/ \!\raisebox{-1ex}{${\mathrm{A}}_{\mathrm{i}}$}\right.}{\raisebox{1ex}{${\mathrm{HR}}_{\mathrm{n}}$}\!\left/ \!\raisebox{-1ex}{${\mathrm{A}}_{\mathrm{n}}$}\right.} $$

HARDi is the health resource agglomeration degree of province i, HRi is the health resource quantity of province I, Ai is the land area of province I, An is the land area of the country, and Pn is the total population of the country. When evaluating the fairness of health resource distribution based on the agglomeration degree, we suggest the following:

1. When the agglomeration degree of health resources is greater than 1, the amount of health resources in the area of the land occupying 1% of the country is greater than 1%, indicating that health resources are relatively more equitable in terms of geographical distribution.
2. When the ratio of HARD and PAD is close to 1, the health resources in this area meet the medical needs of the population, and the residents have better access to health services. If the ratio is greater than 1, it indicates that the health resources in this region are overpopulated; if the ratio is less than 1, it indicates that the resources are insufficient.

## Result

### Equity in the distribution of primary health workforce nationwide

According to the data in 2009 and 2016, there was a considerable increase in all kinds of workforce including health professionals, physicians and nurses, in terms of per 1000 capita and per 1000km^2^. The number of nurses showed fastest growth in terms of per 1000 capita and per 1000km^2^ (Table 2). In terms of regions, the fastest-growing health workforce was in the southern coastal areas and the northwestern zone. However, the Atkinson index showed that the distribution of workforce in China was still close to 1 in general, indicating that the distribution was still relatively unfair (Table 3). The fairness based on population was relatively better than that based on geographical area. The distribution was less fair among nurses. In 2016, its Atkinson index based on population was 0.9632, higher than that for health professionals (0.9320) and physicians (0.9531). Its Atkinson index based on geography was 0.8836, higher than that of health professionals (0.7857) and physicians (0.8506).

**Table 2:** PHC facilities and workforce per capita and per km^2^ in each economic region in 2009 and 2016

Year	Economic zone	Health professionals		Physicians		Nurses	
		Per 1000 capita	Per 1000 km^2^	Per 1000 capita	Per 1000 km^2^	Per 1000 capita	Per 1000 km^2^
2009	Northeast	0.83	0.11	0.42	0.06	0.19	0.03
	Northern coastal	1.07	0.56	0.55	0.29	0.21	0.11
	Eastern coastal	1.19	0.87	0.58	0.42	0.29	0.21
	Southern coastal	0.92	0.40	0.41	0.18	0.27	0.12
	Middle reaches of the Yellow River	1.22	0.14	0.68	0.08	0.22	0.02
	Middle reaches of the Yangtze river	1.21	0.39	0.59	0.19	0.27	0.09
	Southwest	0.16	0.02	0.49	0.09	0.19	0.03
	Northwest	0.91	0.01	0.41	0.01	0.22	0.01
	Total	0.93	0.31	0.52	0.16	0.23	0.08
2016	Northeast	1.43	0.20	0.72	0.10	0.41	0.06
	Northern coastal	1.69	0.97	0.88	0.50	0.46	0.27
	Eastern coastal	2.08	1.58	1.06	0.81	0.63	0.48
	Southern coastal	1.73	0.82	0.80	0.38	0.58	0.27
	Middle reaches of the Yellow River	1.63	0.19	0.80	0.09	0.44	0.05
	Middle reaches of the Yangtze river	1.62	0.54	0.81	0.27	0.49	0.16
	Southwest	1.73	0.31	0.77	0.14	0.51	0.09
	Northwest	1.70	0.03	0.73	0.01	0.52	0.01
	Total	1.70	0.58	0.82	0.29	0.51	0.17

**Table 3:** Trends in Atkinson index based on area and population between 2009 and 2016

Atkinson Index	Year	Health professionals	Physicians	Nurses
Geographic	2009	0.9542	0.9629	0.9757
	2013	0.9349	0.9549	0.9663
	2016	0.9320	0.9531	0.9632
Population	2009	0.8509	0.8803	0.9212
	2013	0.7935	0.8556	0.8930
	2016	0.7857	0.8506	0.8836

### Equity in the national distribution of PHC workforce between urban and rural areas

Table 4 showed the fairness of PHC workforce distribution between urban and rural areas. From the calculation of the Gini coefficient, the fairness for almost all kinds of PHC workforce in the urban and rural areas in China seemed to have improved. Although the distribution of urban physicians had entered a relatively fair state, the fairness of the distributions of health professionals (0.3612–0.3715) and physicians (0.3505–0.3689) in rural areas showed regression. In contrast, the fairness of the three components of the PHC workforce in urban areas was better than that in rural areas, among which the largest gap between urban and rural areas was for physicians; the Gini coefficient was 0.2972 in cities, and 0.3689 in rural areas. The nurses in rural areas had the fastest improvement in fairness (0.3939–0.3673).

**Table 4:** Gini index of PHC workforce by population in urban and rural areas

Year	Health professionals		Physicians		Nurses	
	Urban	Rural	Urban	Rural	Urban	Rural
2009	0.3243	0.3616	0.3284	0.3505	0.3157	0.3939
2013	0.311	0.3573	0.3095	0.3483	0.3055	0.3608
2016	0.3127	0.3715	0.2972	0.3689	0.3124	0.3673

### Equity in the distribution of PHC workforce of eight economic regions

**Fig. 2:**
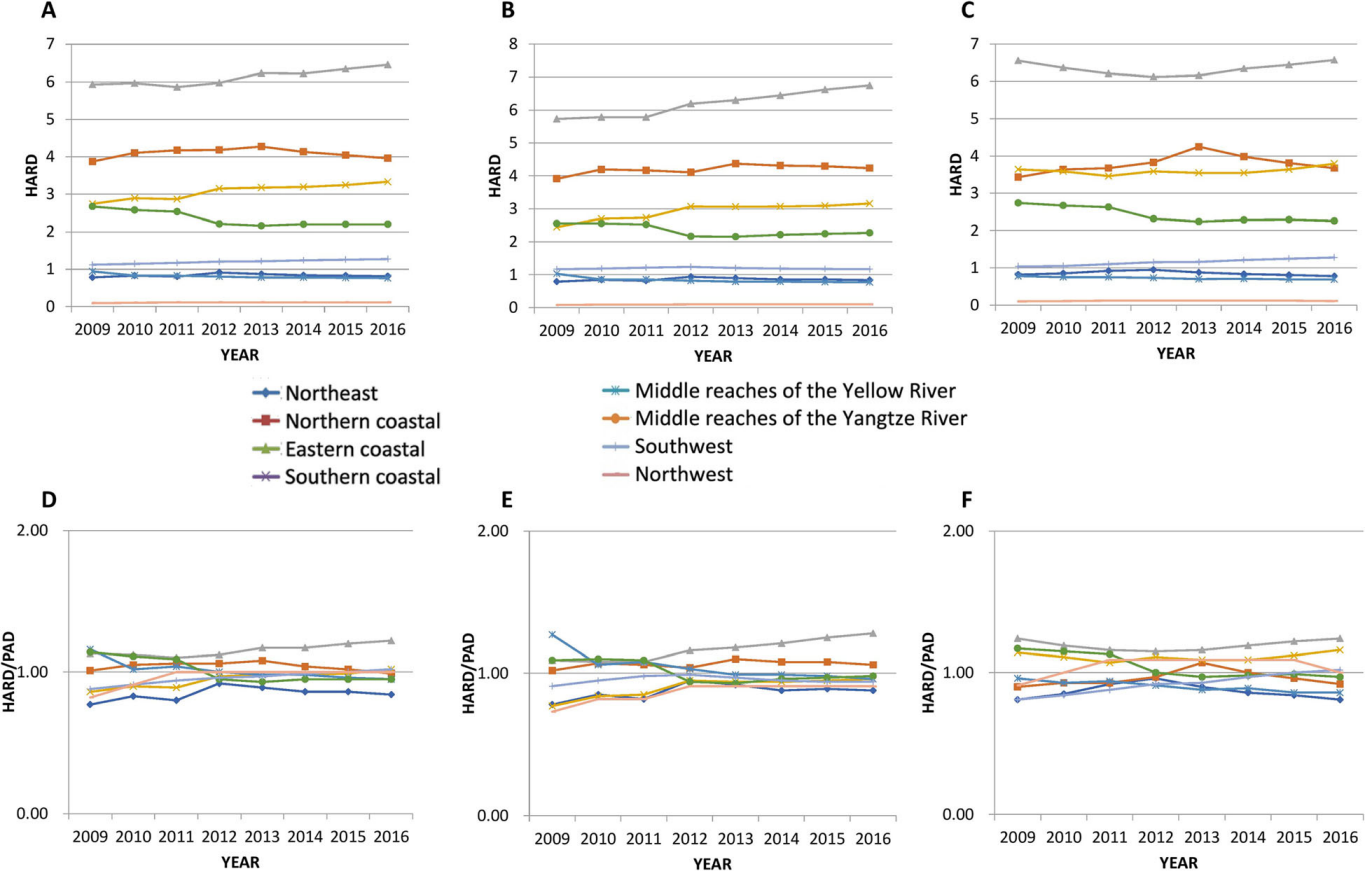
Agglomeration degree of the distinction of PHC workforces in eight economic regions from 2009 to 2016. **a**, **b** and **c** show the proportion of health professionals, physicians and nurses concentrated in a certain land area of 1% of the country, representing the geographical accessibility of PHC workforce allocation. **c**, **d** and **e** respectively. The ratio of the proportion of health professionals, physicians and nurses and the proportion of the population in 1% of the country’s land area in a certain area represents the population accessibility of PHC workforce allocation. The x-axis represents the year, and the y-axis represents the HARD and the HARD/PAD

Figure 2 showed PHC workforce agglomeration degree of eight economic regions. A, B and C showed that the trends on 3 kinds of PHC workforce were similar, and the gaps in resource aggregation degree among the eight regions were large. There existed geographical unfairness for the northwest, southwest, northeast and the middle reaches of the Yellow River. Moreover, except for the southwestern region (HARD increased from 1.12 to 1.27), the agglomeration degree of these regions showed little change during the new medical reforms. D, E and F embodied distributional fairness of PHC workforce over population D and E showed that the aggregation degree trends of health professionals and physicians were similar. From 2009 to 2016, the distributions of the workforce in the middle reaches of the Yangtze River and the middle reaches of the Yellow River changed from surplus to balance, while in the southern coastal region it changed from shortage to sufficient and in the eastern coast it maintained a surplus situation. The other regions were roughly in balance. F showed that the distributional inequality in nurses was more severe than that in health professionals and physicians. The distributional of nurses over geographic area in the northeast and the middle reaches of the Yellow River was still insufficient, while the southern and eastern coasts continued excessive.

### Equity in the distribution of PHC workforce in urban and rural areas in the eight regions

**Fig. 3:**
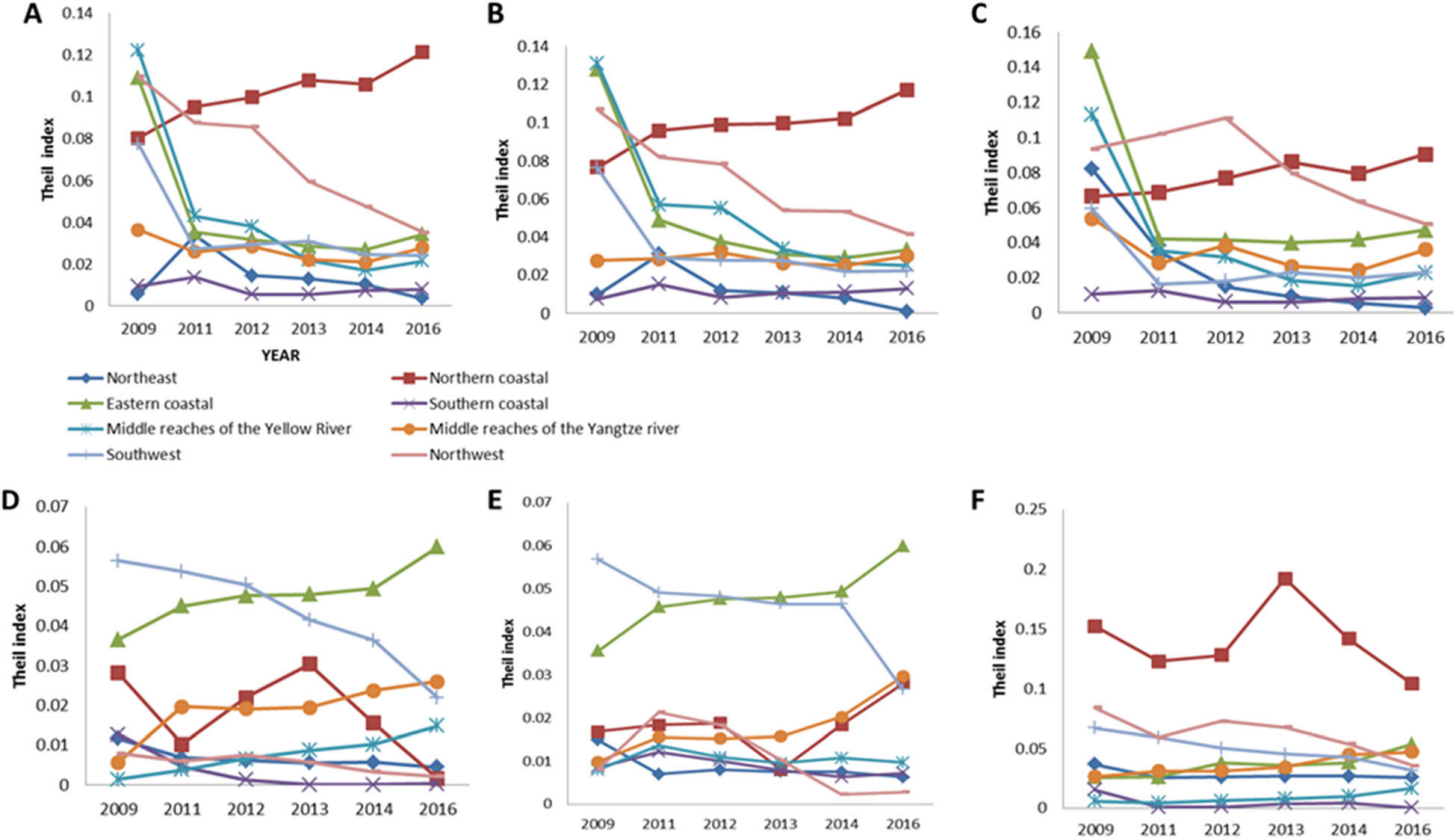
Theil index of the distinction of PHC workforces in urban and rural areas in eight regions from 2009 to 2016. **a**, **b** and **c** show the Theil index of PHC workforces in urban areas of eight economic zones. **c**, **d** and **e** respectively, show the Theil index of PHC workforces in rural areas of eight economic zones. The x-axis represents the year, and the y-axis represents the Theil index

Figure 3 showed the results of the equity analysis in the PHC workforce in urban and rural areas in the eight regions from the Theil index. The distributional fairness of the urban and rural primary health human resources had improved in most areas during the new medical reforms since 2009. A, B and C reflected the trend of fairness for PHC workforce distribution in urban areas of the eight economic zones and indicated that the fairness had improved greatly in all regions except the northern coast. D, E and F reflected the trend of fairness in the rural areas of the eight economic zones. In general, the distributional fairness for nurses was worse than that for physicians and health professionals in rural areas, but had improved after 2013. The timely trends of distributional fairness for health professionals and physicians were similar, and the equity in southwest China was poor in 2009 but had improved significantly in 2016. Notably, the Theil index of the three workforce groups in the rural areas of eastern coastal and the middle reaches of the Yangtze river increased, which indicated that the fairness worsened. The reasons for these trends were worth discussing.

## Discussion

The equity of the entire PHC workforce allocation has generally improved during the new medical reforms. Among the entire PHC workforce, the fairness of the nurse allocation is relatively poor, though improved from 2016 to 2019. Fairness gaps across the eight regions are still large. And the fairness for the entire workforce allocation in the cities has improved, but in rural areas, only the fairness for nurse allocation has improved.

According to the results of the Atkinson index, we can see that the fairness of distribution per person is better than that per unit of geographical area. This is consistent with the findings of other studies on the fairness of health resource distribution in specific regions [30, 31]. It indicates that currently China’s health administrative department allocates PHC resources according to population distribution, while ignoring geographical accessibility [32]. The results suggest that comprehensive consideration based on populational and geographical factors should be incorporated to allocate health resources rationally and scientifically.

Compared with health professionals and physicians, the distribution of nurses was relatively unfair. Increasing studies have shown that the unfairness in configuration of nurses could lead to a decline in patient satisfaction as well as low medical efficiency [33, 34]. Therefore, the government needs to introduce preferential and intensive employment-oriented policies to encourage and guide more students to choose nursing careers, and address the shortage of nurses at the source. At the same time, it is necessary to offer key assistance to regions where nurses are in short supply and to formulate preferential policies to dispatch qualified nurses to these regions in order to minimize the differences in distribution across regions.

In traditional studies, China was usually divided into eastern, central and western regions. Studies on the distribution of health resources concluded that the eastern region was more equitable, followed by the central region, and the western region was the least equitable [7]. However, according to the results of this study, even within the same region, different economic regions had different trends, and the problems they have been facing are also different.

The northern coastal and eastern coastal areas, as pioneers of the reform, took the lead in setting up a pilot site for PHC reform by increasing investment in medical assets and medical-association-owned assets, and attracting qualified healthcare professionals across the country. Such conditions caused the high degree of agglomeration of primary health resources in these areas and the simultaneous resource scarcity in other places. Therefore, it is necessary for the government to establish a mechanism of counterpart support to encourage and guide the flow of health professionals to the areas with resource shortage, such as the northeastern region. In recent years, along with the singularization of industrial structure, the northeast continued to experience an outflow of its young labor, resulting in an accelerated social aging process [35]. The proportion of elderly people increased, while the young labor was insufficient, leading to an imbalance between supply and demand of PHC resource. Therefore, the government needs to establish a positive compensation mechanism to promote the vertical flow of medical resources [36].

In the southern coastal areas, the southwestern areas, the middle reaches of the Yellow River, and the middle reaches of the Yangtze River, fairness has been improved significantly. Although these regions have a large population, their per capita health expenditure accounts for a high proportion of GDP, which shows that all sectors of society attach great importance to PHC and invest a great amount in its development. In addition, local governments formulated many regional strategies, such as the “369 talent project” in the middle reaches of the Yellow River [37].

According to previous studies where China was sectioned into three economic areas, health resources in the western region were always in short supply [38, 39]. However, this study showed that the fairness of workforce in the southwest during the new medical reforms has been greatly improved, while the effectiveness in the northwest has been minimal. Southwestern region has a large population and is mostly in the frontier region. There the local governments actively adopted regional policies. For example, based on full recognition of regional differences, they established a region-specific support system that aimed for high-level hospital support which covered remote and backward areas with comprehensive support in primary institutions, finance and material [40, 41]. Meanwhile, the northwest region is relatively backward and sparsely populated. The fairness of health resource distribution over geographical area is relatively poor. Although the country invested more funds and implemented corresponding talent introduction strategies during medical reforms, due to the lack of comprehensive distribution measures, the situation with poor employment environment, little room for promotion, and high staff turnover remains a major problem among these areas. To avoid the waste of public resources caused by inefficient investment, it is necessary for the government to fully understand the population distribution when allocating health resources, and to deduct the unoccupied areas to more accurately allocate health resources.

Presently, no study has been conducted to compare the fairness in PHC workforce distribution between urban and rural areas during the new medical reforms, and several phenomena that we found in this study are worth discussing. First, the fairness in PHC workforce distribution of both urban and rural areas has improved to varying degrees; the fairness gap between urban and rural areas has been further widened [10]. The socioeconomic development in urban and rural areas was not balanced in China, with workforce surplus in the cities and shortage in rural areas, possibly due to a lack of proper incentive and guiding mechanisms. Second, among all kinds of workforces, the distributional fairness of nurse in rural areas has improved the most. This trend is observed along with a series of preferential policies attracting more nurses to work in rural areas [42]. Third, the fairness for health professional and physician distributions in rural areas has regressed, possibly due to the poor working conditions and the limited development space and training opportunities [43–45]. Fourth, the decline of fairness in some relatively fast-developing areas, such as urban areas of the northern coastal areas, rural areas of the eastern coastal areas, and the middle reaches of the Yangtze River, might be due to the advancement of reform, in which the policies tilted toward the public hospitals, strengthening the “siphon effect” on the health workforce and resources [46, 47].

Agglomeration degree is a new method to measure fairness. In this paper, the agglomeration degree is used to evaluate the equity of PHC resource distribution in China. Currently, relevant studies mainly use the Lorenz curve, the Gini coefficient and the Theil index to evaluate the equity of health resource distribution [35, 36], but these methods are based on the unfairness of the overall evaluation of the region. Although the Theil index distinguishes between inter-regional differences and intra-regional differences, it is impossible to incorporate the combined effects of geographic factors and population distribution in evaluating the fairness of health resource distribution. On the other hand, the agglomeration degree is equivalent to a combination of the Lorenz curve and the Theil index, which makes up for the shortcomings of the two methods when conducting separate evaluations. It considers the influence of population distribution and geographical scale on the fairness of health resource distribution, and the calculation is convenient. Due to its unique advantages, it has been adopted in various fields of health research in recent years [48–50].

## Limitations

This research focused to analyze the fairness of PHC workforce distribution from the perspective of population and geography, without incorporating information on the actual health status and health service needs of people in different regions. At the same time, it focused to analyze quantified healthcare workforce, without checking the scale and service capacity of PHC service institutions.

## Conclusion

Since China started its new medical reforms in 2009, the distribution status of workforce have been continuously optimized. However, there are still some problems.

There is an imbalance of the resource distribution in different regions of the country. The distribution of health resources over population was more equitable than that over geographical areas. The number of nurses increased significantly, but due to the lack of reasonable allocation, the fairness on distribution of nurses was poorer than that of other healthcare human resources. The gap in fairness of the PHC workforce distribution between the urban and rural areas has widened further, and the fairness of the distribution of health professionals and physicians in rural areas shows a trend toward regression.

Currently, China’s medical reforms are still in the stage of exploration and development. In the future, the government should clearly addressed the problems in allocation of health resources and try to narrow the gap in regional financial investment and health expenditure.

## Data Availability

Please contact author for data requests.
